# The Functions and Duties of County Medical Societies

**Published:** 1856-08

**Authors:** 


*The Functions and Duties of County Medical Societies.*—The Preamble to the Act of April 10, 1813 states that, "Whereas well regulated medical societies have been found to contribute to the diffusion of the science, and particularly the knowledge of the healing art: Therefore," etc.

In this preamble we have a general statement of the object of county societies. The fact that only an annual meeting is prescribed by law, and that the distance of the several members from each other must prevent any prolonged stay at the meeting, indicates that other objects must exist for their maintenance than the single purpose of medical improvement, which could hardly be attained by a single annual meeting.

The act following the preamble, in conferring legal rights and disciplinary powers upon the county societies, points out these other objects, and shows them to be the maintenance of a proper police in the profession, the rejection of unworthy candidates for membership, and the promotion of a high professional sentiment. These objects are to be attained in three ways: First, by refusing membership to unworthy men; secondly, by the passage of by-laws to which all members shall be amenable; and third, by the punishment of persons infringing these by-laws.

We propose to consider these three points, premising, however, our belief that in the proper exercise of these functions is comprised the most important duty of county medical societies. Medical improvement, in the scientific sense of the term, is but a secondary object. In the brief sessions prescribed by law, and rendered necessary by the distance of many members from the county seat, in the inability of these members to avail themselves of the advantages of the library, and in the far greater utility, for this purpose, of local and voluntary associations, we find some negative argument for our position; while in the vital necessity which exists of some governing power in the profession, which may heal its differences and decide formally and legally on the conduct of its members, we have a strong positive reason for believing that this latter office constitutes the highest and most peremptory duty of a county society.

Of the three methods by which a county society may maintain its own dignity and character, and enforce its police, the first is found in its power of admission or rejection of candidates for membership.

The power of the society relative to the admission or expulsion of members is derived from the 14th section of the act of 1813, empowering them to make by-laws and regulations "relative to the affairs, concerns and property of said societies, *relative to the admission and expulsion of members*," etc. This power, thus conferred, has been decided to be imperative. The society may withhold membership from whom they please. In the case of Henry G. Dunnel vs. The Medical Society of the County of New York, a motion for a mandamus was made to compel the society to admit the plaintiff. The plaintiff was duly elected a member of the society, but his certificate of membership was refused until he should pay his initiation fee of ten dollars. This he refused to do, and asked for the mandamus.

The Court, Hon. Wm. L. Marcy, Judge, decided that there was no power prohibiting an initiation fee, and that as the power is given to county societies "to establish by-laws relative to the admission of members," the plaintiff had no remedy. The effect of this decision is to give to any county society the power to reject any applicant not fully complying with their by-laws, and this power is of course delegated to those officers who perfect the admission of a member, after the vote authorizing his admission "on complying with the by-laws."

So far as its effect upon the applicant is concerned, the conditions are best expressed in the opinion rendered by Hon. Greene C. Bronson to a committee of the S ate Medical Society, Feb. 6, 1833.

"*First.* Every physician, not already a member, must apply for admission into the society of his county, on pain of the forfeiture of his license.

"*Second.* The society, in pursuance of its by-laws and regulations, may either admit or reject the applicant. If rejected, he loses nothing but the benefits which he might have enjoyed from the association. His license is not forfeited."

It will be seen from the above that the power of the societies relative to admission is ample.

The second object, that of the maintenance of a proper police in its membership, is reached through the same general power, conferred by statute (Section 14 of Act of 1813) to make by-laws and regulations. This power is very general in its terms. It does not dictate as to particulars, but merely provides that nothing shall be enacted contravening the laws of the State, or its constitution. Within this broad limit the society has ample range for the government of its members. It holds the power of infamous expulsion through the courts, depriving the person expelled of all license to practice; and it has, again, another form of expulsion which, without the intervention of the courts, simply deprives the member of the benefits of the association. It will readily be seen that a member may be expelled under the latter power, who could not be under the former. For instance, a society, under its power to make by-laws and regulations, may declare a certain act dishonorable to its members, which is not dishonorable aside from that connection. It does this on the principle that the good of community, or of the society, demands such a concession of the individual rights of the member. Thus, as in the case of "The People vs. The Society of the County of New York," a pecuniary sacrifice of ten dollars was demanded, and the society was sustained by the court. Again the society has power to declare certain conduct, such as the practice of quackery, dishonorable, and may expel for it; although no one can dispute the legal right of any man to practice homœopathy, or manufacture and vend secret remedies.

This power, then, enables county societies to decide (with a view to the general interests of the profession) that acts, in themselves legal, shall, nevertheless, be considered dishonorable, and to punish them by expulsion or otherwise. Such was the action of the Erie County Medical Society in expelling John D. Hill on the charge of dishonorable conduct, in accepting an office at a price less than that dictated by their tariff. This action was justified, inasmuch as it had been proven to and decided by the society, that too great cheapness in services to the county had resulted disastrously both to the poor and the honor of the profession. But does not such action of the society violate that principle of law which forbids the combination of a number of men to extort certain prices by compulsory measures?

The position of the society is this. We deem it important to the interests of the poor, and to the honor of the profession, that each member should in this instance, as in private practice, forego the natural right of underbidding. Any member who has so little regard for the poor, or the profession, as to refuse to do so, is guilty of dishonorable conduct. We do not deny his legal right to underbid, but we claim the right to clear our skirts of any part of the dishonor by depriving him — not of his license — but of his privilege of association with us. To compel us to associate with him, would be depriving us of our natural right to choose our own company.

It is evident that the county society has no power to deprive a member of his license, without the intervention of the courts. The Hon. Greene C. Bronson, in the opinion before quoted, states that, "The society, in pursuance of its by-laws and regulations, may either expel a member, or may deliver charges to the District Attorney: and the member may be expelled the society by the judges of the county court. In the first case the member loses nothing but the benefits of the association; in the last case, he may be forever disqualified from pursuing his profession, or be suspended from its practice for a limited period."

It seems, then, that in the third function mentioned, that of punishment, there are, aside from votes of censure, etc., two forms of expulsion:

In the first form the county society acts alone, and is governed as to method not by the act of 1813, but by its own by-laws. These in the case of the Erie County Medical Society provide that any member may be expelled by a vote of a majority of the members present. This form of expulsion by resolution has been four times resorted to, in as many different cases.

What should be the form of trial? In the first place, it should be recollected that the society has no power to summon witnesses, or compel them to testify. This at once does away with the ordinary forms of trial. Again, who are the judges, who the jury, and who the witnesses? Any member of the society may combine all three functions. What, then, is the true position of a society in this form of expulsion?

It has been decided in the courts of Oneida county, that no form of trial is necessary, the society standing toward the accused member in the same position as a judge when dealing peremptorily with a person guilty of contempt of court. In both these cases there is no trial by jury, and yet it is not held to contravene the Bill of Rights.

Finally, what should be the form of trial through the medium of the courts?

As this involves the deprivation of a license, only infamous acts should be tried in this method, and the form of trial requires written and specific charges, ten days notice in the public prints, and a day specially set apart for the trial. The society, thus convened, have power to expel by resolution, two-thirds voting aye, and then must send their charges to the District Attorney. If the county court confirm the action of the society, the member is deprived of the right to practice—if it refuses to confirm that action, the member is nevertheless expelled, though he does not forfeit his license.

In the case occurring in Oneida Co., a member was accused of procuring an abortion, was tried therefor by the county society, and only a majority voting for expulsion, he retained his membership. He was subsequently indicted for the same offence, when, on trial, it was proven that he procured the abortion, but, the child not being quick, he was, under the then existing laws, acquitted. After this acquittal, the county society expelled him by a two-thirds vote, and sent charges to the district attorney. It was held by the court that his previous acquittal did not hinder the action of the society, and that in so expelling him they were not creating a crime unknown to law, neither were they acting in an extra-judicial capacity, that the expulsion by resolution was sufficiently formal, the society acting like a court in a case of contempt, that the court had no power to compel the society to receive him again to membership; and, finally, the court confirmed the action of the society, and deprived him of his license to practice medicine.

